# Recombination and Positive Selection Differentially Shaped the Diversity of *Betacoronavirus* Subgenera

**DOI:** 10.3390/v12111313

**Published:** 2020-11-16

**Authors:** Diego Forni, Rachele Cagliani, Manuela Sironi

**Affiliations:** Scientific Institute IRCCS E. MEDEA, Bioinformatics, 23842 Bosisio Parini, Italy; rachele.cagliani@lanostrafamiglia.it (R.C.); manuela.sironi@lanostrafamiglia.it (M.S.)

**Keywords:** coronavirus, virus evolution, recombination, positive selection, betacoronavirus, genome evolution

## Abstract

The *Betacoronavirus* genus of mammal-infecting viruses includes three subgenera (*Sarbecovirus*, *Embecovirus,* and *Merbecovirus)*, in which most known human coronaviruses, including SARS-CoV-2, cluster. Coronaviruses are prone to host shifts, with recombination and positive selection possibly contributing to their high zoonotic potential. We analyzed the role of these two forces in the evolution of viruses belonging to the *Betacoronavirus* genus. The results showed that recombination has been pervasive during sarbecovirus evolution, and it is more widespread in this subgenus compared to the other two. In both sarbecoviruses and merbecoviruses, recombination hotspots are clearly observed. Conversely, positive selection was a less prominent force in sarbecoviruses compared to embecoviruses and merbecoviruses and targeted distinct genomic regions in the three subgenera, with S being the major target in sarbecoviruses alone. Overall, the results herein indicate that *Betacoronavirus* subgenera evolved along different trajectories, which might recapitulate their host preferences or reflect the origins of the presently available coronavirus sequences.

## 1. Introduction

The members of the *Coronaviridae* family (order *Nidovirales)* are enveloped, positive-sense, single-stranded RNA viruses infecting three classes of vertebrates: mammals, birds, and fish. Coronaviruses have long and complex genomes, unusual if compared to those of other RNA viruses. A large portion of the coronavirus genome encodes two large, overlapping open reading frames (ORF1a and ORF1b) that are translated and processed into 16 non-structural proteins (nsp1 to 16) [1]. The remaining portion of the genome encodes structural proteins—spike (S), envelope (E), membrane (M), and nucleoprotein (N)—as well as a variable number of accessory proteins [1,2,3]. Several coronavirus genera and subgenera are recognized (https://talk.ictvonline.org/ictv-reports/). In particular, the *Betacoronavirus* genus includes five out of the seven coronaviruses known to infect humans [4,5,6]. Two “common cold” coronaviruses, human coronavirus OC43 and human coronavirus HKU1, are members of the *Embecovirus* subgenus, whereas MERS-CoV (Middle East respiratory syndrome coronavirus) is a member of the *Merbecovirus* subgenus [3]. The recently emerged human-infecting coronavirus, now referred to as SARS-CoV-2 [6] and responsible for the recent pandemic, clusters with SARS-CoV (severe acute respiratory syndrome coronavirus) and other bat-derived viruses in the *Sarbecovirus* subgenus [6,7,8]. 

In general, bats host a large diversity of coronaviruses. Both SARS-CoV and MERS-CoV originated in bats and were transmitted to humans via an intermediate host [2,3]. Most likely, SARS-CoV-2 also originated and evolved in bats, eventually spilling over to humans, either directly or through an intermediate, unknown host [7,9,10,11,12,13]. Generally, coronaviruses are prone to cross-species barriers, resulting in a high zoonotic potential [14].

Recombination and positive selection are major evolutionary forces driving viral genome evolution and possibly contributing to host jumps. Complex recombination events have played a role in the frequent host shifts that occurred during coronavirus evolutionary history [3,15,16,17,18]. The S gene was a major target of these events, possibly because the spike protein has a central role in the interaction with the host, in terms both of cell entry and of immune evasion [3,15,16]. Additionally, the emergence of new mutations that confer an advantage in infecting and efficiently spreading in a new host are usually maintained by the action of positive selection [3,19].

Herein, we analyzed the evolution of the *Betacoronavirus* genus using available genomic data for sarbecoviruses, merbecoviruses, and embecoviruses. Our aim was to determine the relative contribution of recombination and positive selection in driving the evolution of *Betacoronavirus* subgenera.

## 2. Materials and Methods

### 2.1. Sequences and Alignments

Genome sequences were retrieved from the National Center for Biotechnology Information database (NCBI, http://www.ncbi.nlm.nih.gov/). The genome sequence of RmYN02 was downloaded from the GISAID initiative website (https://www.gisaid.org).

All the available complete genomes of sarbecoviruses, merbecoviruses, and embecoviruses were retrieved, but only sequences sampled in different hosts and with less than 99% nucleotide identity in pairwise comparisons were included in the analysis (Table S1). Pairwise identity scores were calculated as 1-(M/N), where M is the number of mismatching nucleotides and N is the total number of positions along the alignment at which neither sequence has a gap or an undetermined character.

Alignments were generated using MAFFT [20], setting the sequence type as nucleotide or codons, as appropriate.

### 2.2. Recombination Analysis

Evidence for recombination signals in each *Betacoronavirus* subgenus was searched for using the 3SEQ software (v.1.7) [21]. This tool tests all sequence triplets in a given alignment, scanning for mosaic recombination signals. The result is the identification of mosaic regions in which one of the three sequences is the recombinant (child) of the other two (parental). To be conservative, a significance threshold of 0.01 was applied. This method was selected because it has very good power in detecting recombination events, and it is faster than most other approaches [21], thus allowing comparison among datasets by resampling.

To compare the frequencies of recombination in different viral subgenera, we counted the numbers of bona fide unique recombination events. We defined bona fide unique events as all those events that involved the same region in the subgenus alignment, with identical genomic positions for both the start and the end of the recombination segments. We also considered as bona fide unique events all those events with breakpoints falling in a range of 100 nucleotides both from the beginning and from the end of the recombination segments.

### 2.3. Detection of Positive Selection in Betacoronavirus Subgenera

Coronavirus ORFs were independently analyzed for the presence of positive selection signals. 

Because recombination can inflate positive selection analyses, the 3SEQ results were taken into account. In particular, each ORF sequence was divided into subregions based on the genomic locations of the recombination breakpoints (if any), so as to obtain non-recombinant regions. Only regions longer than 500 (for ORF1a/ORF1b) or 100 (for all other ORFs) nucleotides falling between two recombination breakpoints (or the start/stop codon and a breakpoint) were considered for the analysis (Table 1, Table 2 and Table 3). The E gene was excluded from the analysis due to its low dS in the sarbecovirus lineage [22]. For the same reason, and due to the presence of RNA secondary structures, two N regions and one ORF3a region were masked [22]. ORF10 was not analyzed, as most sarbecoviruses do not encode the full-length protein [22].

To compare the level of positive selection among subgenera, embecovirus and merbecovirus alignments were divided based on sarbecovirus division. For each ORF, we considered the number and the codon length of regions defined in the sarbecovirus analysis, and we generated the same number of regions of the same length for the other two subgenera (Table 1, Tables S2 and S3). Clearly, we also took into account the distribution of the recombination breakpoints in each subgenus; thus, some regions did not exactly match in terms of codon length.

Phylogenetic trees were generated with the phyML software (v3.1), by applying a General Time Reversible (GTR) model with gamma-distributed rates, 4 substitution rate categories, and the estimation of the transition/transversion ratio and proportion of invariable sites [23].

Episodic diversifying selection was analyzed by using the aBSREL (adaptive Branch-Site Random Effects Likelihood [24]) method implemented in the HYPHY suite (version 2.5) [25]. aBSREL was run by testing whether a proportion of the sites of each internal branch of the phylogeny had evolved under positive selection. A Holm–Bonferroni-corrected *p* value of ≤0.05 for the likelihood ratio test was considered as evidence of statistical significance. To avoid false-positive signals deriving from sequencing errors and transient mutations, tip branches were excluded from the analysis.

## 3. Results

### 3.1. Recombination Plays a Major Role in Sarbecovirus Evolution

**Figure 1:**
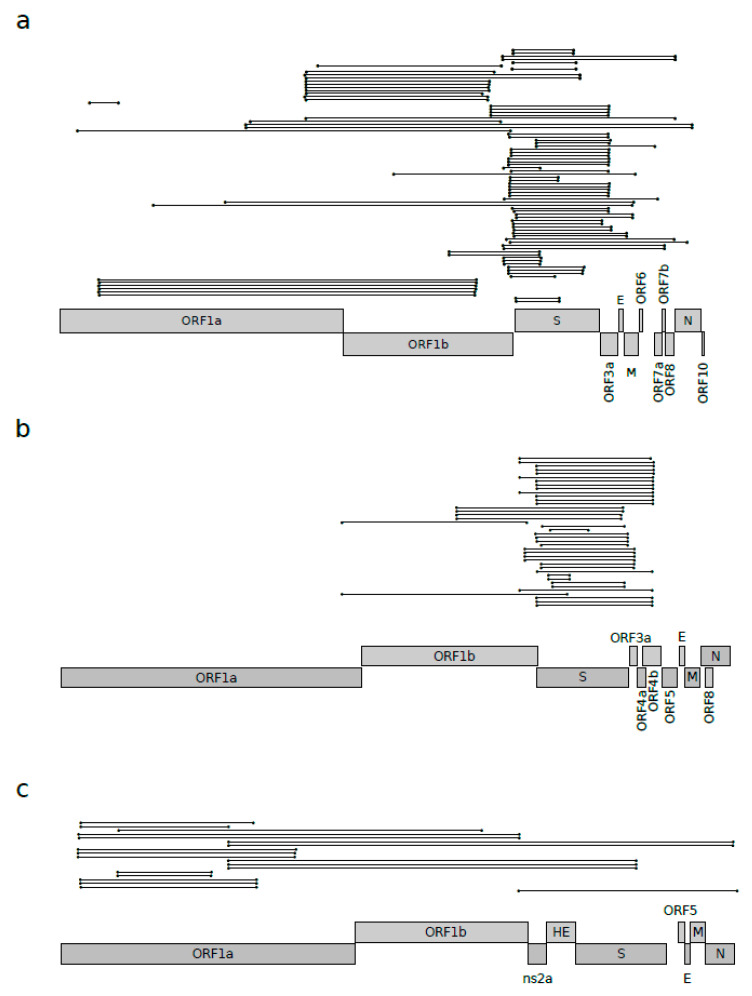
Recombination events in *Betacoronavirus* subgenera. Unique recombination events identified by 3SEQ in (**a**) sarbecovirus, (**b**) merbecovirus, and (**c**) embecovirus alignments. Each recombination event is shown as a line with dots representing the start and the end. Unique events were defined as those having exactly the same breakpoint positions. Schematic representations of ORF positions are also reported for SARS-CoV-2 in panel **a**, MERS-CoV in panel **b**, and HCoV-OC43 in panel **c**.

Recombination is known to play a major role in the evolution of coronaviruses [3,15,16,17,18]. For this reason, we aimed to quantify the amount of recombination responsible for the shaping of known sarbecovirus genomes. We thus used the 3SEQ software [21] to estimate the number of recombination events acting on a genome alignment of 46 sarbecoviruses (Table S1). These were selected to be representative of the viral subgenus and for having less than 99% identity according to pairwise comparisons. The results highlighted the presence of several recombinant segments (92 events), scattered along the genome, with a high proportion of events involving the genomic region encompassing the S and ORF3a genes and, to a lesser extent, ORF1b (Figure 1a).

We next evaluated whether these events had a major effect in the evolution of sarbecoviruses and if recombination occurs with similar frequencies in other *Betacoronavirus* subgenera. We thus carried out the same analysis for viruses in the *Embecovirus* and *Merbecovirus* subgenera, and we compared the frequencies of recombination events. Using the same selection criteria described above, fifteen merbecoviruses were analyzed (Table S1), and the 3SEQ analysis showed that, for this subgenus, recombination is less pervasive, both in terms of the number of events (44 recombination events) and, more markedly, in terms of the genomic distribution (Figure 1b). In fact, in merbecoviruses, almost all the events were located in the region surrounding the spike protein (Figure 1b). The same approach was applied to 15 embecoviruses (Table S1); in this case, 3SEQ analysis showed even fewer recombination events (25 events), most of them condensed in the ORF1ab region (Figure 1c).

To formally test whether the *Sarbecovirus* subgenus experienced more recombination events compared to the other two subgenera, we counted the number of bona fide unique recombination events identified for the three subgenera. In particular, bona fide unique events were defined as events that involved the same genomic region, with identical or very similar (less than 100 nucleotides apart) positions for both the start and the end of the recombination segments (Figure 1a). Using this approach, we found 39 bona fide unique events for sarbecoviruses, 15 events for merbecoviruses, and 10 for embecoviruses.

Clearly, differences in the number of events may derive from the higher number of analyzed sarbecovirus strains. We thus randomly selected 15 sarbecovirus strains, and we ran a 3SEQ analysis. We repeated this analysis 100 times, and we counted the number of bona fide unique recombination events for each of them. The results showed that in 85 out of 100 cases, the sampled sarbecoviruses had at least 15 bona fide unique recombination events, suggesting that recombination is a major component of sarbecovirus evolution and that it is more widespread compared to that for the *Merbecovirus* and *Embecovirus* subgenera. However, the sarbecovirus dataset is not only larger than the merbecovirus and embecovirus datasets, but it is also biased in terms of host representation, as several viruses were sampled from *Rhinolophus sinicus* (20 out of 46 genomes). For recombination to occur, the same animal must be infected by two (or more) parental viruses. This is clearly more likely to happen for viruses that infect the same host and may explain the higher frequency of recombination in sarbecoviruses. To partially account for this effect, we repeated the 3SEQ analysis using a subset of 13 sarbecoviruses sampled from different hosts. Using this sample, we identified 16 bona fide unique recombination events. The random sampling of 13 merbecoviruses or embecoviruses indicated that in very few cases (0 out of 100 for embecoviruses and 5 out of 100 for merbecoviruses) are 16 or more bona fide unique recombination events detected. This analysis suggests that the higher recombination frequency in the *Sarbecovirus* subgenera is not only determined by the sampling bias. Overall, the latter seems to play a minor role in the overall estimate of the recombination frequency. In fact, the random sampling of 13 sarbecoviruses among those isolated from *Rhinolophus sinicus* identified a similar number (15) of bona fide unique recombination events to those detected in genomes sampled by different hosts. It should nonetheless be noted that several hosts belong to the *Rhinolophus* genus (Table S1).

### 3.2. Positive Selection Acting on Betacoronaviruses

**Figure 2:**
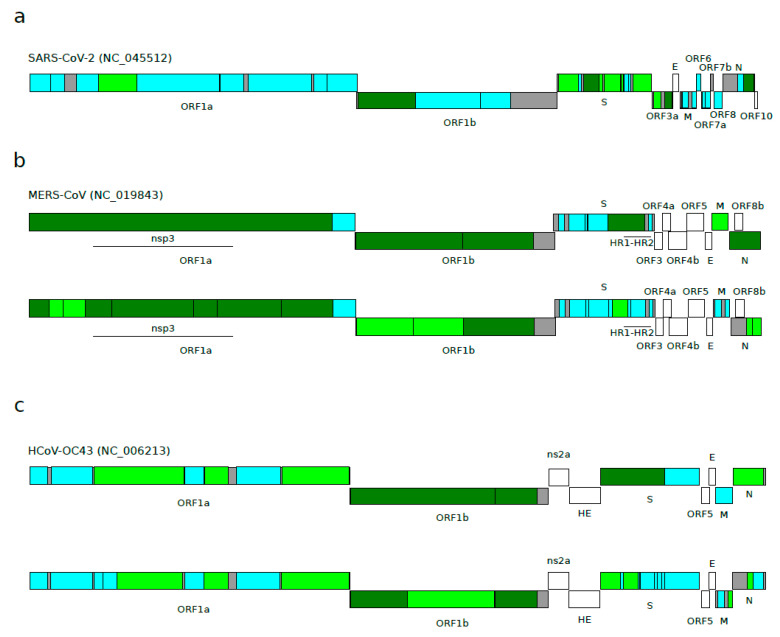
Episodic diversifying selection in *Betacoronavirus* subgenera. Schematic representation of positive selection signals detected by aBSREL in (**a**) sarbecoviruses, (**b**) merbecoviruses, and (**c**) embecoviruses. A representative viral species of each lineage was used to map ORFs and non-recombinant regions. Color codes are as follows: cyan, region analyzed with no signal of positive selection; light green, region analyzed with 1 branch of the phylogeny under positive selection; dark green, region analyzed with at least 2 branches of the phylogeny under positive selection; gray, regions not analyzed due to the presence of many breakpoint events; white, regions excluded *a priori* from the analyses. See methods for details regarding region selection. Merbecovirus and embecovirus alignments were divided based on their 3SEQ results (panels **b** and **c**, upper schemes), and also based on their 3SEQ results and sarbecovirus 3SEQ results (panels **b** and **c**, lower schemes) so as to obtain regions of similar lengths to in sarbecoviruses. Merbecovirus regions found under positive selection in previous studies [45,49] are also reported. HR1-HR2: heptad repeat domains 1 and 2.

We next aimed to assess whether positive selection has been driving the evolution of sarbecoviruses. Indeed, recombination and positive selection are both responsible for generating genomic diversity, but positive selection can be over-estimated in the presence of recombination [26]. To overcome this problem, we analyzed each sarbecovirus ORF after taking into account the recombination results described above. Positive selection, conventionally defined as a higher non-synonymous substitution rate (dN) than expected based on the rate of synonymous substitutions (dS), was estimated using the aBSREL (adaptive Branch-Site Random Effects Likelihood) method. aBSREL relies on branch-site models to test if positive selection has occurred on a proportion of branches in a phylogeny. The E gene was excluded from the analysis because we have previously shown that the gene has unusually low dS, most likely due to the presence of a conserved RNA secondary structure (see the methods section for details regarding the region selection) [22]. Taking into account recombination signals, we generated 31 non-recombinant regions, and we searched for evidence of diversifying positive selection in all the internal branches of the sarbecovirus phylogeny (Table 1). Out of the 31 regions analyzed, 11 showed at least one internal branch under positive selection (35.4%) (Figure 2a and Figure S1, Table 1). A closer inspection revealed that six out of eight regions in the S gene display evidence of positive selection, whereas only two regions (out of 11) were found to be under positive selection for ORF1ab. The other three regions were located in the N (1) and ORF3a (2) genes (Figure 2a and Figure S1, Table 1).

Again, we wanted to verify whether episodic positive selection occurs at a different frequency in sarbecoviruses compared to other *Betacoronavirus* subgenera. Using the same approach applied for sarbecoviruses, we analyzed 12 merbecovirus and 13 embecovirus coding regions, encompassing ORF1ab, S, M, and N (Table 2 and Table 3). Accessory proteins were excluded from the analysis because they differ in number among betacoronaviruses and cannot therefore be compared. For six merbecovirus regions, at least one branch experienced the action of positive selection (50%), and the selected branches were almost all located in the ORF1ab, M, and N genes (Figure 2b and Figure S2, Table 2). For the embecovirus alignment, seven regions were found to be under positive selection (53.8%), and five of them were located in the ORF1ab gene (out of nine regions) (Figure 2c and Figure S3, Table 3).

All together, these results suggest that positive selection is not more common in sarbecoviruses compared to other betacoronaviruses. However, the different numbers of analyzed regions (i.e., 31 for sarbecoviruses compared to the 12 and 13 for viruses in the other two subgenera) and their different lengths could have biased the results we obtained. Thus, we divided the merbecovirus and embecovirus alignments into the same number of regions as the sarbecovirus ORF1a, ORF1b, S, M, and N regions (i.e., 25 regions), with similar alignment lengths, taking into account the recombination signals specific for each subgenus (Tables S2 and S3).

For the merbecovirus alignment, we found 14 regions with at least one positively selected branch (56%) (Figure 2b, Table S2), whereas for embecoviruses, we found 10 regions as positively selected (40%) (Figure 2c, Table S3). The analysis of the same regions in the sarbecovirus viruses had identified nine regions (36%) (Figure 2).

When we investigated single ORFs, we found that ORF1a and ORF1b showed a strong level of selection for merbecoviruses (11 regions out of 12 identified as positively selected) and embecoviruses (six regions selected), whereas only two regions were found as positively selected for sarbecoviruses (Figure 2). An opposite scenario emerged when the S gene was considered. The sarbecovirus analysis found six out of nine regions to be under positive selection, whereas merbecovirus and embecovirus presented only one and three regions, respectively (Figure 2).

Overall, these data indicate that positive selection tends to be a less prominent force in the evolution of sarbecoviruses compared to the other two *Betacoronavirus* subgenera. Additionally, the main target of positive selection in sarbecoviruses is the spike protein, with almost no signals in the long polyprotein.

## 4. Discussion

Here, we analyzed the evolution of the genomes of viruses belonging to different *Betacoronavirus* subgenera with the aim of assessing the relative importance of two evolutionary forces, namely, recombination and positive selection. Recombination was previously shown to be pervasive in sarbecoviruses and, in general, in coronaviruses [2,3,18]. For instance, SARS-CoV emerged from recombination events among bat-hosted coronaviruses [17,27,28,29], and MERS-CoV originated from the exchange of genetic material among viruses isolated from camels and bats [30]. As for SARS-CoV-2, different recombination events were described, with a particular focus on the receptor-binding domain of the spike protein. Indeed, SARS-CoV-2 shows a receptor-binding motif (RBM) nearly identical to that of the pangolin viruses, which instead differs from the sequence of its known closest relative (i.e., the bat virus RaTG13). Whereas this clearly suggests that ancestral recombination events had a role in the evolution of SARS-CoV-2 and related viruses, the incomplete sampling of bat sarbecoviruses makes it difficult to reconstruct such events [12,18,31,32].

Our results confirm that recombination has been driving the evolution of viruses in the three *Betacoronavirus* subgenera. However, by using a resampling approach to correct for the different numbers of sequences and for the host bias, we found that recombination is more common in sarbecoviruses compared to embecoviruses and merbecoviruses. We note that, although we accounted for possible biases, this result may not necessarily derive from different features of betacoronavirus genomes. In the case of sarbecoviruses, most sequences were obtained from Asian bats and several were obtained from a Yunnan cave, where different bat species roost [29]. Thus, even if we restrict analysis to viruses that were isolated from different hosts, most of such hosts are bats living in nearby geographic areas. It is thus more likely that, for these viruses, both the parentals and the recombinants were sampled, eventually increasing the power for detecting recombination. Conversely, merbecoviruses’ and embecoviruses’ genomes were obtained from more diverse hosts, in terms of both taxonomy and of geographic origin. Thus, ancestral recombination events or events with unsampled parentals may have been missed by the 3SEQ analysis.

In line with recent data [18], we found several recombination events along the sarbecovirus genome alignment, with a few clear recombination hotspots involving the terminal portion of ORF1b, and the boundary between ORF1b and the S gene, as well as ORF3. In principle, the clustering of recombination breakpoints might reflect epistatic interactions across the sarbecovirus genomes—i.e., that a fraction of recombinant viruses have reduced fitness due to the loss of interactions among co-evolved sites. This was previously shown for animal and plant viruses [33,34,35,36,37,38,39]. For instance, fewer breakpoints than expected are located within the *env* gene of HIV-1, whereas, in ssDNA viruses, recombination breakpoints tend to fall either outside genes or at their edges [38,39]. Indeed, in line with previous results [18], most breakpoints we detected in sarbecoviruses and merbecoviruses were located at either side of S, suggesting that the spike-coding sequence is frequently transferred as a unit from one genetic background to another. However, several breakpoints fell within the S gene, as well. In sarbecoviruses, this was previously observed and related to the emergence of viruses with specific sequence features in the RBM [18,31]. Moreover, in sarbecoviruses, we detected recombination breakpoints within other structural (M and N) and non-structural proteins (including ORF3a, ORF8, and several nsps), suggesting that the disruption of intra-genome interactions is not a major driver of breakpoint clustering. It should nonetheless be noted that data from other viruses indicated that secondary recombination events or mutations can restore high fitness to defective viruses originating from unfavorable primary recombination events [37]. An alternative possibility for the clustering of recombination breakpoints is that specific features (the base composition and presence of secondary structures) favor recombination events [38,39,40,41].

Recently, Li and coworkers reported that sarbecovirus coding sequences evolved under strong purifying selection [31]. Indeed, this is a general feature for most viruses (and cellular organisms, as well) [19,42,43]. The pervasive action of purifying selection does not exclude the possibility, however, that a fraction of sites evolve under different regimes and possibly do so only on specific branches of a phylogeny. We thus searched for evidence of episodic positive selection in betacoronaviruses by applying the aBSREL model, which tests whether a proportion of sites are positively selected on one or more branches (the internal branches in this case) of a phylogeny. Specifically, aBSREL does not specify a priori the number of omega classes for each branch but infers it probabilistically. Thus, aBSREL is well-suited to analyzing regions of different sizes and branches of different lengths, which may display very different evolutionary patterns. We only analyzed the internal branches of the phylogenies because, compared to external branches, they are expected to be less affected by sequencing errors and to contain fewer polymorphic sites or transient substitutions.

Quantitatively, we found that episodic positive selection was less pervasive in sarbecoviruses compared to merbecoviruses and embecoviruses, possibly suggesting that the former viruses rely more on recombination than on positive selection to generate adaptive diversity. The distribution of positively selected regions was also very different among subgenera. In sarbecoviruses, the S gene and ORF3a were the major targets of episodic positive selection, with most regions within these genes showing one or more positively selected branches. Specifically, within S, the RBD showed evidence of selection on three branches. Overall, these results are in agreement with a recent report of episodic positive selection in sarbecoviruses [44]. Conversely, positive selection in merbecoviruses and embecoviruses mainly targeted ORF1a and ORF1b, in line with previous results in a smaller merbecovirus phylogeny [45]. For these viruses, limited evidence of episodic positive selection was detected for structural proteins. One possible explanation for the stronger positive selection in merbecoviruses and embecoviruses compared to sarbecoviruses may again reside in the wider range of hosts from which these viruses were sampled. Host shifts in other RNA viruses have been associated with adaptive changes in several proteins other than the structural ones. For instance, the adaptation of avian flu viruses to mammals is often characterized by changes in the viral polymerase and other non-structural proteins [46,47,48]. It is thus possible that signals of episodic positive selection in merbecovirus and embecovirus ORF1a/ORF1b genes represent signatures of adaptation to non-bat hosts.

In summary, our results indicate that different evolutionary forces have been acting with different strengths on *Betacoronavirus* subgenera. Clearly, the continuous sampling of different animal coronaviruses is of pivotal importance for gaining insight into the genetic diversity of these viruses in animals and to promote surveillance strategies for potential zoonoses.

## Figures and Tables

**Table 1:** Analysis of episodic diversifying selection in sarbecovirus open reading frames (ORFs).

Sarbecovirus			
ORF	Region	Alignment Length	aBSREL Result
ORF1a			
	reg1	834	0 branches under selection among 43 tested
	reg2	570	0 branches under selection among 43 tested
	reg3	897	0 branches under selection among 43 tested
	reg4	1653	1 branch under selection among 43 tested
	reg5	3393	0 branches under selection among 43 tested
	reg6	963	0 branches under selection among 43 tested
	reg7	2568	0 branches under selection among 43 tested
	reg8	537	0 branches under selection among 43 tested
	reg9	1221	0 branches under selection among 43 tested
ORF1b			
	reg1	2316	2 branches under selection among 43 tested.
	reg2	2613	0 branches under selection among 43 tested
	reg3	1212	0 branches under selection among 43 tested
S			
	reg1	843	1 branch under selection among 43 tested
	reg2	141	0 branches under selection among 43 tested
	reg3	624	2 branches under selection among 43 tested
	reg4	141	1 branch under selection among 42 tested
	reg5	648	1 branch under selection among 43 tested
	reg6	114	1 branch under selection among 43 tested
	reg7	183	0 branches under selection among 43 tested
	reg8	114	0 branches under selection among 43 tested
	reg9	750	1 branch under selection among 43 tested
ORF3a			
	reg1	291	1 branch under selection among 43 tested
	reg2	315	3 branches under selection among 43 tested
M			
	reg1	261	0 branches under selection among 43 tested
	reg2	171	0 branches under selection among 42 tested
ORF6		183	0 branches under selection among 42 tested
ORF7a			
	reg1	126	0 branches under selection among 42 tested
	reg2	216	0 branches under selection among 43 tested
ORF8			
	reg1	378	0 branches under selection among 40 tested
N			
	reg1	234	0 branches under selection among 41 tested
	reg2	444	2 branches under selection among 43 tested

**Table 2:** Analysis of episodic diversifying selection in merbecovirus ORFs.

ORF	Region	Alignment Length	aBSREL Result
ORF1a			
	reg1	12,960	7 branches under selection among 12 tested
	reg2	927	0 branches under selection among 12 tested
ORF1b			
	reg1	4314	4 branches under selection among 12 tested
	reg2	2889	3 branches under selection among 12 tested
S			
	reg1	279	0 branches under selection among 12 tested
	reg2	654	0 branches under selection among 12 tested
	reg3	108	0 branches under selection among 12 tested
	reg4	849	0 branches under selection among 12 tested
	reg5	1512	3 branches under selection among 12 tested
	reg6	150	0 branches under selection among 12 tested
M		663	1 branch under selection among 12 tested
N		1341	6 branches under selection among 12 tested

**Table 3:** Analysis of episodic diversifying selection in embecovirus ORFs.

ORF	Region	Alignment Length	aBSREL Result
ORF1a			
	reg1	723	0 branches under selection among 12 tested
	reg2	1731	0 branches under selection among 12 tested
	reg3	4419	1 branch under selection among 12 tested
	reg4	783	0 branches under selection among 12 tested
	reg5	987	1 branch under selection among 12 tested
	reg6	1800	0 branches under selection among 12 tested
	reg7	2775	1 branch under selection among 12 tested
ORF1b			
	reg1	5919	3 branches under selection among 12 tested
	reg2	1740	3 branches under selection among 12 tested
S			
	reg1	2823	2 branches under selection among 12 tested
	reg2	1449	0 branches under selection among 12 tested
M		693	0 branches under selection among 12 tested
N	reg1	1299	1 branch under selection among 12 tested

## Supplementary Materials

The following are available online at https://www.mdpi.com/1999-4915/12/11/1313/s1, Table S1. List of viral strains analyzed in this study. Table S2. Analysis of episodic diversifying selection in merbecovirus ORFs. Table S3. Analysis of episodic diversifying selection in embecovirus ORFs. Figure S1. Episodic diversifying selection in sarbecoviruses. Figure S2. Episodic diversifying selection in merbecoviruses. Figure S3. Episodic diversifying selection in embecoviruses.
